# Development of a Magnetic Nanoparticles-Based Screen-Printed Electrodes (MNPs-SPEs) Biosensor for the Quantification of Ochratoxin A in Cereal and Feed Samples

**DOI:** 10.3390/toxins10080317

**Published:** 2018-08-06

**Authors:** Xian Zhang, Zuohuan Wang, Hui Xie, Renjie Sun, Tong Cao, Narayan Paudyal, Weihuan Fang, Houhui Song

**Affiliations:** 1 China-Australian Joint Laboratory for Animal Health Big Data Analytics, Zhejiang Provincial Engineering Laboratory for Animal Health Inspection and Internet Technology, College of Animal Science and Technology, Zhejiang A&F University, Lin’an 311300, China; zhangxian073@163.com (X.Z.); whfang@zju.edu.cn (W.F.); 2 Zhejiang University Institute of Preventive Veterinary Medicine & Zhejiang Provincial Key Laboratory of Preventive Veterinary Medicine, 388 Yuhangtang Road, Hangzhou 310058, China; zuohuanwang@zju.edu.cn (Z.W.); rjsun@zju.edu.cn (R.S.); caotong0915@zju.edu.cn (T.C.); narayan.paudyal@outlook.com (N.P.); 3 Zhejiang Key Laboratory of Experimental Animal and Safety Evaluation, Zhejiang Academy of Medical Sciences, Hangzhou 310013, China; xhui828@163.com

**Keywords:** ochratoxin A, magnetic nanoparticles, screen-printed electrodes, quantification

## Abstract

A rapid and sensitive electrochemical biosensor based on magnetic nanoparticles and screen-printed electrodes (MNPs-SPEs sensor) was developed for the detection of ochratoxin A (OTA) in cereal and feed samples. Different types of magnetic nanoparticles-based ELISA (MNPs-ELISA) were optimized, and the signal detection, as well as sensitivity, was enhanced by the combined use of screen-printed electrodes (SPEs). Under the optimized conditions, the calibration curve of the MNPs-SPEs sensor was *y* = 0.3372*x* + 0.8324 (*R*^2^ = 0.9805). The linear range of detection and the detection limit were 0.01–0.82 ng/mL and 0.007 ng/mL, respectively. In addition, 50% inhibition (IC50) was detectable at 0.10 ng/mL. The limit of detection (LOD) of this MNPs-SPEs sensor in cereal and feed samples was 0.28 μg/kg. The recovery rates in spiked samples were between 78.7% and 113.5%, and the relative standard deviations (RSDs) were 3.6–9.8%, with the coefficient of variation lower than 15%. Parallel analysis of commercial samples (corn, wheat, and feedstuff) showed a good correlation between MNPs-SPEs sensor and liquid chromatography tandem mass spectrometry (LC/MS-MS). This new method provides a rapid, highly sensitive, and less time-consuming method to determine levels of ochratoxin A in cereal and feedstuff samples.

## 1. Introduction

Ochratoxins are secondary metabolites with toxic properties, produced by some species of fungi including *Penicillium* and *Aspergillus* and could be easily found in moldy or fermented agricultural products. The most ubiquitous and toxic of the ochratoxins is ochratoxin A (OTA). OTA can be present in various agricultural products, food, and feedstuffs when they are harvested, processed, stored, or transported in improper conditions [1,2]. Mycotoxins are nephrotoxic, teratogenic, carcinogenic, and immunosuppressive with serious health risks to animals, as well as humans [3,4]. The International Agency for Research on Cancer has classified OTA as a possible human carcinogen (group 2B) [5]. The European Commission (EC No. 123/2005) has set maximum tolerated OTA levels, which state that the OTA concentration should not exceed 5 ppb in cereals, 2 ppb in wine and juices, and 0.5 ppb in infant food [6]. To minimize risk, a detection technique that is easy to operate and yet possesses a high sensitivity is preferred for accurately determining the levels of OTA in food and feeds.

At present, the technology of OTA analysis mainly involves traditional analytical techniques, such as thin-layer chromatography (TLC) [7], high-performance liquid chromatography (HPLC), gas chromatography (GC), and liquid chromatography tandem mass spectrometry (LC-MS/MS) [8,9,10]. Although these methods have good accuracy and reproducibility, the requirements of professional operators, expensive equipment, cumbersome sample pretreatment processes, or long processing times have limited their practical applications. Thus, alternative approaches, such as lateral flow strips, enzyme- or fluorescent-linked immunosorbent assay, and surface plasmon resonance (SPR) biosensors, have been developed [10,11]. Each of these systems has their own advantages; for example, lateral flow strips are relatively cheaper, can be used onsite, and the results can be observed with naked eyes or with a portable densitometric analyzer. Enzyme-linked immunosorbent assays (ELISAs) are rapid and accurate, and SPR biosensors can be performed in real time [12,13,14,15]. These methods are largely popular for a quick detection of multiple analytes. Despite such popularities, such methods are less sensitive (and may not be suitable for trace contaminants), thus limiting their usage. Receptor alternatives to antibodies, such as aptamers and molecularly imprinted polymers, also have been utilized for a wide variety of applications [16,17]. Aptasensor, an emerging method of detection, has attracted more attention, due to its high sensitivity, selectivity, and simplicity.

In recent years, magnetic nanoparticles (MNPs), owing to their uniform diameter particles that can be homogenously distributed in colloidal suspension, have been used widely in biomedical and food-safety assays [18,19,20]. When the magnetic nanoparticles (MNPs) are used for the preparation of immunomagnetic beads, a covalent bond binds the nanoparticle with the antibody to form a complex. Thus, the formed complex binds with the antigens present in the test solution, which is then segregated by a magnetic field. Enhanced sensitivity within a shorter time and the liquid physical state of the reaction medium are the advantages of this method [21]. Highly sensitive, magnetic nanoparticles and biotin/streptavidin-based ELISA (MNPs-bsELISA) was previously reported for rapid detection of zearalenone (ZEN) by our laboratory [22]. A combination of magnetic nanoparticles-conjugated antibodies and a biotin–streptavidin system accentuates the signal detection and thus the sensitivity of the assay. However, there is a need of an additional incubation step to lengthen the reaction time when utilizing the biotin–streptavidin system for signal amplification. Electrochemical sensors used to detect traces of various kinds of analytes respond to specific analytes by converting the signal generated by a chemical reaction to an electrical signal [23,24,25,26,27]. However, the use of the electrode surface as a solid phase for antibody immobilization, as well as electrochemical transducer could result in a reduced electrochemical signal [28].

**Figure 1:**
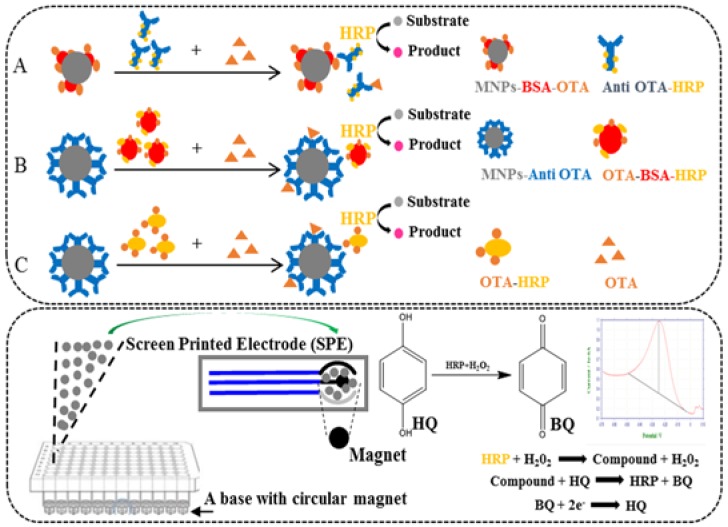
Schematic illustrations of the three different types of magnetic nanoparticles-based ELISA (MNPs-ELISA) (top) and magnetic nanoparticles and screen-printed electrodes-based electrochemical biosensor (MNPs-SPEs sensor) (bottom). (**A**) Anti OTA-HRP MNPs-ELISA, (**B**) OTA-BSA-HRP MNPs-ELISA, and (**C**) OTA-HRP MNPs-ELISA.

This study outlines the development of a rapid and sensitive assay based on magnetic nanoparticles and screen-printed electrodes (MNPs-SPEs sensor) for the detection of ochratoxin A. A scheme of this MNPs-SPEs sensor is shown (Figure 1). Firstly, we established three different types of magnetic nanoparticles-based ELISA (MNPs-ELISA). All the analytical parameters and variables for each method were optimized to increase the sensitivities of the MNPs-ELISA. Then, we explored the use of screen-printed electrodes (SPEs) for the transduction step, coupled with the MNPs-ELISA method to trace the amounts of OTA. This new MNPs-SPEs sensor provides a rapid and accurate approach for the detection of OTA in naturally contaminated samples.

## 2. Results and Discussion

### 2.1. Optimizations and Comparisons of Three Variants of MNPs-ELISA

The dilutions of MNPs-anti OTA, MNPs-BSA-OTA, concentration of OTA-BSA-HRP, anti OTA-HRP, and OTA-HRP of the three types of MNPs-ELISA were optimized, and the results are shown in Table 1. The time for the competition reaction of each MNPs-ELISA was 45 min.

The limit of detection (LOD), 50% inhibitory concentration (IC50), and detection ranges of the three different types of MNPs-ELISAs were calculated using Microsoft Excel (version 2016) (Table 2) under the optimized conditions.

**Figure 2:**
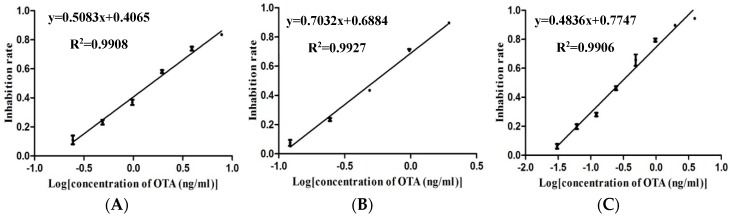
Calibration curves of different types of MNPs-ELISA. The log concentration of ochratoxin A is plotted along the *x*-axis, while the inhibition rate is on the *y*-axis. The error bar indicates the standard deviation. (**A**) Anti OTA-HRP MNPs-ELISA, (**B**) OTA-BSA-HRP MNPs-ELISA, and (**C**) OTA-HRP MNPs-ELISA.

The LOD is defined as the average signal corresponding to three standard deviations from the signals of mycotoxin-free samples [29], whereas the concentration of target mycotoxin causing 20–80% inhibition is the detection range [30]. The sensitivities, as well as the working ranges, were improved when using the immune-magnetic beads methods (Figure 1B) compared with the antigen-coated magnetic nanoparticles (Figure 1A) and improved even further when using the OTA-HRP (Figure 1C). This result is consistent with the previous study, which showed that for the detection of small-molecule analytes, the conjugation of analytes with HRP can be used for signal amplification [31]. The three calibration curves are presented in Figure 2.

### 2.2. Specificity Study

The MNPs-ELISA based on MNPs-anti OTA and OTA-HRP had low cross-reactivity with the OTA analogue-OTB (5.7%), which was consistent with our previous study (anti-OTA-based ELISA) [32]. No cross-reactivities were observed with other different mycotoxins, including AFB_1_, FB_1_, ZEN, PAT, CIT, and DON (<0.01%). These results demonstrated the good specificity of this MNPS-ELISA for the detection of OTA.

### 2.3. Optimization of the Electrochemical Biosensor Immunoassay

For the electrochemical test, several parameters were optimized to improve the performance. Different concentrations of H_2_O_2_ and hydroquinone (HQ) concentrations were assayed after the recognition event, where only tracer is added (0 OTA ppb). The use of 1mM of H_2_O_2_ and 1.5 μM of HQ resulted in an increased electrochemical signal. The optimum scanning frequency was −0.5 to −0.1 V and a scan speed of 100 mV s^−1^.

**Figure 3:**
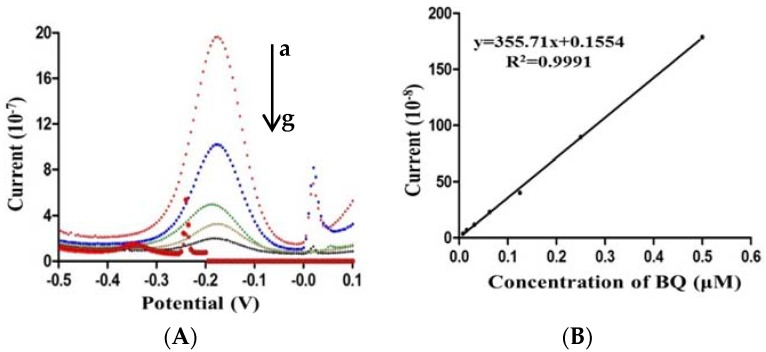
(**A**) Differential pulse voltammetry (DPV) scanning curve using multiple concentrations of benzoquinone (BQ) ranging from 0.5 μM (the highest peak, denoted by a) to the lowest of 0.078 μM (denoted by g) and (**B**) the linear relationship between peak currents and BQ concentrations.

The differential pulse voltammetry (DPV) scanning curve of benzoquinone (BQ) with different concentrations (seven units of twofold serial dilution from 0.5 μM) and the linear relationship between peak currents and concentrations are shown in Figure 3, which permitted the development of the electrochemical analytical method based on the catalytic conversion of HQ for the detection of OTA.

### 2.4. Calibration Curve of the Electrochemical Biosensor Immunoassay

**Figure 4:**
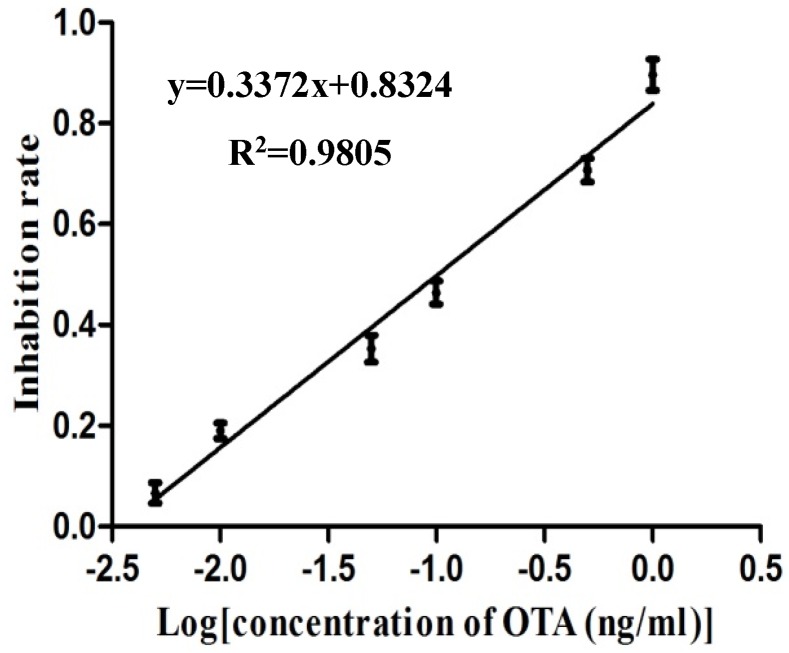
Calibration cure for quantification of ochratoxin A by magnetic nanoparticles-based electrochemical immunosensor (MNPs-SPEs sensor). The error bar indicates the standard deviation.

The calibration curve of the electrochemical biosensor immunoassay is shown in Figure 4. The equation of linearity is *y* = 0.3372*x* + 0.8324 with *R*^2^ of 0.9805. The range of detection and the detection limit were 0.01–0.82 ng/mL and 0.007 ng/mL, respectively. In addition, 50% inhibition (IC50) was detectable at 0.10 ng/mL.

Previous studies have reported that in the detection of small-molecule analytes, a conjugation of analytes with HRP can be used for signal amplification [31]. Magnetic nanoparticle (MNPs), owing to their uniform diameter particles that can be homogenously distributed in colloidal suspension, reduced detection time and improved sensitivity. In this study, a MNPs-ELISA was developed using MNPs-anti OTA and OTA-HRP for signal amplification. As the electrochemical analysis has proven its ability in detecting traces of various analytes, a rapid and sensitive biosensor based on the MNPs-ELISA and screen-printed electrodes (MNPs-SPEs sensor) was established. By converting the signal generated by a chemical reaction to an electrical signal, the signal amplification was achieved once again. The detection limit (0.007 ng/mL) of the MNPs-SPEs sensor was significantly better than MNPs-ELISA (0.04 ng/mL), and the sensitivity increment was 10 old when compared with a common ELISA (0.07 ng/mL) established in our laboratory [32].

This presented MNPs-SPEs sensor offers higher sensitivity as compared with other methods in the detection of OTA, including fluorescence methods [33,34,35], enzyme immunoassay [30], fluorescence polarization (FP) analysis [36], and other different sensor methods [37,38,39,40,41]. Compared with other ultrasensitive techniques, such as fluorescence resonance energy transfer (FRET) aptasensor [42] and impedimetric aptasensor [43], our MNPs-SPEs sensor offers a similar detection limit, but in a shorter detection time. The comparative results of the performance parameters of these detection methods are shown in Table 3. The easy to use MNPs-SPEs sensor described herein can give precise and accurate results in a short time. This detection method is suitable not only for mycotoxins, but also can be modified for other analytes, such as antibiotics, drugs, and pesticide residues.

### 2.5. Recovery Studies

Corn samples spiked with OTA at different levels (i.e., 1.25, 2.5, 5, 10, and 20 μg/kg) were analyzed in triplicate using the MNPs-SPEs sensor, and the results are shown in Table 4. The recovery rates were between 78.7% and 113.5%, and the relative standard deviations (RSDs) were 3.6–9.8%, which indicates that this MNPs-SPEs sensor assay was accurate and has good reproducibility.

### 2.6. Commercial Samples Analysis

**Figure 5:**
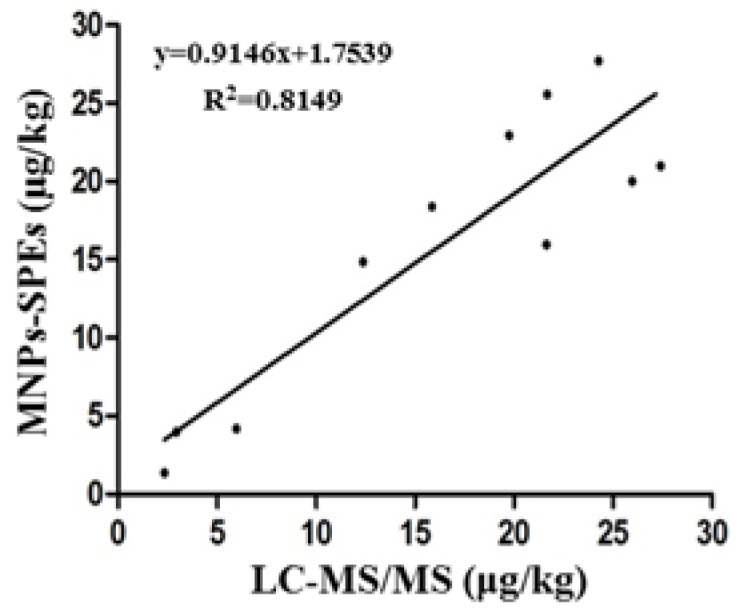
Regression analysis of results of ochratoxin A levels detected by MNPs-SPEs sensor and liquid chromatography tandem mass spectrometry (LC-MS/MS).

A total of 56 dry commercial samples of corn, wheat, and feedstuff were analyzed in triplicate by the MNPs-SPEs sensor method and LC-MS/MS. The results of the positive samples are shown in Table 5. Using these 11 samples (5 corn, 4 wheat, and 2 feed) that were positive for ochratoxin, the relationship estimates between the MNPs-SPEs sensor and LC-MS/MS methods for OTA detection was assessed by regression analysis, MNPs-SPEs sensor = 1.7539 + 0.9146LC-MS/MS (*R*^2^ = 0.8149). It revealed that there is a good agreement between these two methods (Figure 5). This also demonstrate that our MNPs-SPEs sensor system is applicable for the detection of OTA.

## 3. Conclusions

In this work, a screen-printed electrodes (SPEs)-based electrochemical biosensor (MNPs-SPEs sensor) was successfully engineered for the detection of ochratoxin A in cereal and feed samples utilizing magnet nanoparticles (MNPs) and a small molecule-HRP conjugate (OTA-HRP) for signal enhancement. For MNPs-SPEs sensor systems, the detection limit was 0.007 ng/mL, the detection range was 0.01–0.82 ng/mL, and the IC50 was 0.10 ng/mL. The recovery rates in spiked cereal samples spread between 78.7–113.5%, and the RSD values were all <15%. Analysis of commercial samples using this MNPs-SPEs sensor and LC-MS/MS revealed a good correlation between these two methods. The lower limit of detection and shorter reaction time of less than one hour makes this new assay an excellent alternative to existing conventional methods for the detection of trace amounts of OTA in agroproducts. Additionally, this platform can also be adapted for the detection of other small targets, as well as offering wide applications in food safety-related fields.

## 4. Materials and Methods

### 4.1. Materials

Analytical standards of mycotoxins, bovine serum albumin (BSA), and peroxidase from horseradish (HRP) were obtained from Sigma-Aldrich (St. Louis, MO, USA). The OTA monoclonal antibody 2D8 (mAb, 2D8) was prepared in our laboratory [32]. The activated peroxidase protein labeling kit (EL0156) was purchased from Huzhou InnoReagents Co., Ltd. (Huzhou, China). Magnetic nanoparticles (M-270, 14305D) were purchased from Invitrogen (Carlsbad, CA, USA). Screen-printed electrodes based on carbon working and counter electrodes were provided by Prof. P. Wang, Biosensor National Special Laboratory, Zhejiang University. Other reagents of analytical grade, including hydrogen peroxide (30%) (H_2_O_2_), hydroquinone (HQ), and benzoquinone (BQ) were purchased from Sinopharm Chemical Reagent Co. Ltd. (Shanghai, China).

### 4.2. Equipment

The equipment used in this study were the following: the 37 °C incubator from Thermo Scientific (Waltham, MA, USA); the horizontal shaker (Vortex 4 basic) from IKA (Staufen, Germany); the magnetic separator (MS-12) from Bangs laboratories (Fishers, IN, USA); and the Spectra Max M_2_ micro-plate reader from Molecular Devices (Sunnyvale, CA, USA). Electrochemical measurements were performed with a PC-controlled CHI-832 electrochemical analyzer (Chenhua, Shanghai, China).

### 4.3. Synthesis of the OTA and Antibody Conjugates

OTA-HRP was prepared by a two-step approach with slight modifications [44]. OTA (2.0 mg) was dissolved in 350 mL of anhydrous tetrahydrofuran (THF) and then 3.0 mg of *N*-hydroxy-succinimide (NHS) and 12.0 mg of *N*,*N*′-dicyclohexylcarbodiimide (DCC) were added, followed by gentle shaking at room temperature (RT) for 12 h. The reaction mixture was centrifuged (10,000× *g*, 15 min). The supernatant was dried and dissolved in 0.3 mL dimethyl sulfoxide (DMSO).

HRP (2 mg) was dissolved in 2.0 mL of 0.13 M phosphate buffer (PBS, pH 8.0), and the activated OTA (0.3 mL) was added drop-wise. The reaction was proceeded by vigorous shaking at RT for 6 h and then dialyzed extensively against 0.01 M/L of PBS (pH 7.4) at 4 °C. The OTA-BSA conjugate was synthesized as previously reported [32]. In brief, OTA was dissolved in THF and NHS and DCC were added, followed by gentle shaking at room temperature (RT). The reaction mixture was centrifuged, the supernatant was dried, and the residue was dissolved in DMSO. BSA was dissolved in 0.13 M phosphate buffer (pH 8.0). The activated OTA was added drop-wise to the BSA solution. The reaction was allowed to proceed by vigorous shaking at RT and then dialyzed extensively against 0.01 M/L of phosphate buffered saline (PBS, pH 7.4). OTA-BSA-HRP and Anti OTA-HRP were synthetized as recommended by the supplier (EL0156, Huzhou InnoReagents, Huzhou, China).

### 4.4. Preparation of Magnetic Nanoparticles Conjugates

The immunomagnetic beads (MNPs-Anti OTA) were synthetized and the coating efficiency was measured as described in our previous study [22]. OTA-BSA-coated magnetic nanoparticles (MNPs-BSA-OTA) were prepared in similar fashion as the immunomagnetic beads. In brief, OTA-BSA (100 μg) in coating buffer (100 μL) was added to the activated magnetic nanoparticles and incubated for 2 h at 4 °C with slow tilting rotation. After quenching, the non-reacted activated carboxylic acid groups with quenching buffer, the coated nanoparticles were resuspended in the storage buffer for later use. The coating/quenching/storage buffers were the same as those mentioned in the preparation of MNPs-Anti OTA.

### 4.5. Development of MNP-ELISAs

Three different types of MNPs-ELISA were developed using the magnetic nanoparticles to assay their effects on signal release and performance. Figure 1 shows an illustration comparing these MNPs-ELISA variants.

As shown in Figure 1A, MNPs-BSA-OTA diluted with the storage buffer (10 μL), 70 μL of the Anti OTA-HRP solution, and 70 μL of the diluted test sample extracts (or OTA standard solution) were added to the 96-well plate in triplicate wells. Then, shaking the plate at 1000 rpm for 45 min at 37 °C was followed by placing the samples on a magnetic base to segregate the nanoparticles. The immunomagnetic nanoparticle complexes thus separated were washed thrice. The substrate 3,3′,5,5′-tetramethylbenzidine (TMB,100 μL) was added. Stop solution (2 M H_2_SO_4_, 50 μL) was pipetted after 10 min of rotatory incubation at 37 °C, and OD450 was read on a Spectra Max M2 micro-plate reader. The calibration curve for MNPs-bsELISA was prepared in GraphPad 5 software with the *x*-axis representing the log concentration of OTA (ng/mL) and the *y*-axis, the inhibition rate. The inhibition rate is calculated as one minus the ratio of the OD450 of standard OTA solutions in PBS to the OD450 of PBS (0.01 M, pH 7.4).

As shown in Figure 1B, compared with Figure 1A, the variant involved replacing MNPs-BSA-OTA with MNPs-Anti OTA. For the subsequent steps, diluted sample extracts or standard OTA solutions were mixed with an equal volume of OTA-BSA-HRP (70 μL), and then, the mixtures (150 μL) were transferred to the 96-well plate. Other steps were similar to those described in Figure 1A.

In Figure 1C, as compared with Figure 1B, OTA-BSA-HRP was replaced with OTA-HRP. For downstream assay, diluted sample extracts or standard OTA solutions were mixed with an equal volume of OTA-HRP (70 μL), and then, the mixtures (150 μL) were transferred to the 96-well plate, with other steps similar to those described in Figure 1A.

### 4.6. Optimization of MNPs-ELISAs

Performance enhancement of these three types of MNPs-ELISA was obtained by optimizing the dilutions of MNPs-Anti OTA, MNPs-BSA-OTA, concentrations of OTA-BSA-HRP, Anti OTA-HRP, and OTA-HRP, and times for the competition reaction were as described in our previous study [22]. The concentrations/dilution ratios of immune-reagents for different MNPs-ELISAs were optimized by checkerboard titration design with an OD450 value of about 1.0. Incubation times for the competition reaction were 30, 45, 60, and 90 min.

### 4.7. Specificity Study

To evaluate the specificity of MNPs-ELISA, cross-reactivities (CRs) with seven other different mycotoxins (OTB, AFB_1_, FB_1_, ZEN, DON, PAT, and CIT) were determined, as reported previously [22]. In brief, using the developed MNPs-bsELISA, the calibration curve of OTA with different concentrations was established first. Then, different concentrations of each analyte (instead of OTA) were used as potential binding competitors, and the calibration curve including the IC50 (50% inhibition) for each analyte was calculated respectively. Cross-reactivity was calculated as percent inhibition using the following formula: IC50 of OTA/IC50 of other mycotoxins × 100% [32].

### 4.8. Development of the Electrochemical Biosensor Immunoassay

Electrochemical biosensor immunoassay is developed based on the MNPs-ELISA. After the competition reaction of the MNPs-ELISA is complete, immunomagnetic nanoparticle complexes were resuspended in 100 μL of PBS buffer and transferred onto the surface of a screen-printed electrode on which nanoparticles were immobilized by placing a magnet at the bottom. The immobilized complexes were then rinsed thoroughly with ultrapure water and dried with nitrogen. After that, 100 μL of a solution containing the enzymatic substrate (1 mM H_2_O_2_) and the electrochemical mediator (1.5 μM HQ) in PBS buffer (50 mM) was deposited on the electrode surface with volume enough for covering the three-electrode system. The current response was measured using differential pulse voltammetry (DPV). All DPV measurements were performed in the potential range of −0.5 to −0.1 V and a scan speed of 100 mV s^−1^. The extent of affinity reaction was evaluated by the addition of an electrochemical mediator whose reduction on the electrode surface is directly related to the activity of the enzyme tracer. Figure 1 shows the schematic illustration of the magnetic nanoparticles-based electrochemical biosensor (MNPs-SPEs sensor) test procedure.

### 4.9. Recovery Study and Comparison of Detection in Commercial Samples by LC-MS/MS

OTA-free corn samples (tested by LC-MS/MS) were ground and dried by overnight incubation in a 60 °C incubator and spiked with a standardized solution of OTA at different concentrations. Then, the spiked samples were vortexed for 10 min and incubated at RT overnight. Next, 25 mL of methanol/water (70:30, *v*/*v*) were added to each sample (5 g) and vortexed for 10 min. The samples were centrifuged at 3000g for 10 min, and the supernatants were diluted seven-fold with PBS to minimize the influence of the solvents. Each sample was analyzed in triplicate.

Dry commercial samples (including corn, wheat, and feedstuff) were analyzed by the developed MNPs-SPEs sensor and LC-MS/MS in parallel. Each sample was tested in triplicate to calculate the standard deviation. For the detection by the electrochemical immunoassay, the samples were extracted as the spiked samples. Validated procedures for LC-MS/MS were adopted as those described previously [45].

The correlation between these two methods was calculated using linear regression (Microsoft Excel software, Redmond, WA, USA; 2016 version).

## Figures and Tables

**Table 1:** Concentrations/dilution ratios of immunoreagents for the three different MNPs-ELISAs.

Analytical Methods	Final Concentration (μg/mL)			Dilution Ratio	
	Anti OTA-HRP	OTA-BSA-HRP	OTA-HRP	MNPs-BSA-OTA	MNPs-Anti OTA
Anti OTA-HRP MNPs-ELISA	0.2	NU ^a^	NU	1/50	NU
OTA-BSA-HRP MNPs-ELISA	NU	0.1	NU	NU	1/100
OTA-HRP MNPs-ELISA	NU	NU	0.5	NU	1/100

^a^ NU: Not Used.

**Table 2:** Comparison of the performance attributes of the three different MNPs-ELISAs.

Analytical Method	LOD (ng/mL)	IC_50_ (ng/mL)	Detection Range (IC_20_–IC_80_, ng/mL)	Regression Equation
Anti OTA-HRP MNPs-ELISA	0.25	1.53	0.39–5.94	*y* = 0.5083*x* + 0.4065 (*R*^2^ = 0.9908)
OTA-BSA-HRP MNPs-ELISA	0.14	0.54	0.20–1.44	*y* = 0.7032*x* + 0.6884 (*R*^2^ = 0.9927)
OTA-HRP MNPs-ELISA	0.04	0.31	0.06–1.13	*y* = 0.4836*x* + 0.7477 (*R*^2^ = 0.9906)

**Table 3:** Comparison of the performance parameters of MNPs-SPEs sensor and various other methods for OTA detection.

Methods	Matrix	LOD (ng/mL)	Recovery (%)	Detection Range (ng/mL)	Ref.
Nanobody-based ELISA	Cereal	0.16	80–105	0.27–1.47	[30]
dsDNA-base fluorescence method	Corn	5	89.2–94.1	0.00–100	[33]
Portable Flurescence	Cocoa	1.25	79.05–83.25	1.25–10	[34]
Silver nanoparticles-based LFIA	Juice and wine	0.06	88.0–110.0	0.08–5.0	[35]
Fluorescence polarisation aptamer	Wine	1.1	83–113	-	[36]
Quantum dots-based aptasensor	Foodstuff	0.5	-	1–30	[37]
Fluorescent biosensor	Corn	2.57	96.5–101.4	5.0–160	[38]
A Label-Free Aptasensor	Corn	0.012	96–106	0.04–0.48	[39]
A Polyaniline film-based aptasensor	-	0.1	-	0.1–10	[40]
Fluorescent aptamer-based sensor	Corn	0.8	83–106	1–100	[41]
FRET aptasensor	Peanut	0.0025	90–110	0.01–20	[42]
Impedimetric aptasensor	Wine	0.002	102–107	0.002–6	[43]
MNPs-SPEs sensor	Cereal and feed	0.007	78.7–113.5	0.01–0.82	This work

- not mentioned.

**Table 4:** Recovery and coefficient of variances for different concentrations of ochratoxin A from spiked corn samples.

Samples	Concentrations (μg/kg)		Recovery Rate (%) (Mean ± SD ^a^)	CV ^b^ (%)
	Spiked	Detected		
1	1.25	1.42	113.5 ± 3.6	3.2
2	2.5	1.97	78.7 ± 7.3	9.3
3	5	4.26	85.3 ± 9.8	11.5
4	10	8.12	81.2 ± 5.8	7.2
5	20	18.34	91.6 ± 7.4	8.1

^a^ SD: Standard Deviation (*n* = 3). ^b^ CV: Coefficient of Variation.

**Table 5:** Mycotoxin levels in the commercial samples as determined *by the new MNPs-SPEs sensor and LC-MS/MS*.

Samples	MNPs-SPEs Sensor (μg/kg), Mean ± SD ^a^	LC-MS/MS (μg/kg), Mean ± SD
Corn 1	12.37 ± 1.86	14.87 ± 1.21
Corn 2	19.75 ± 2.17	22.94 ± 1.32
Corn 3	21.67 ± 1.69	25.57 ± 2.01
Corn 4	2.94 ± 0.67	3.98 ± 0.73
Corn 5	24.28 ± 2.41	27.71 ± 2.13
Wheat 1	27.41 ± 1.61	20.99 ± 1.71
Wheat 2	25.97 ± 2.03	20.01 ± 1.61
Wheat 3	2.35 ± 0.97	1.36 ± 0.12
Wheat 4	5.98 ± 1.03	4.18 ± 0.31
Feedstuff 1	15.86 ± 1.15	18.39 ± 1.03
Feedstuff 2	21.65 ± 1.24	15.97 ± 1.41

^a^ SD: Standard Deviation (*n* = 3).
